# Inferring Functional Epigenetic Modules by Integrative Analysis of Multiple Heterogeneous Networks

**DOI:** 10.3389/fgene.2021.706952

**Published:** 2021-08-24

**Authors:** Zengfa Dou, Xiaoke Ma

**Affiliations:** ^1^The 20-th Research Institute, China Electronics Technology Group Corporation, Xi'an, China; ^2^School of Computer Science and Technology, Xidian University, Xi'an, China

**Keywords:** DNA methylation, network biology, functional epigenetic module, non-negative matrix factorization, heterogeneous network

## Abstract

Gene expression and methylation are critical biological processes for cells, and how to integrate these heterogeneous data has been extensively investigated, which is the foundation for revealing the underlying patterns of cancers. The vast majority of the current algorithms fuse gene methylation and expression into a network, failing to fully explore the relations and heterogeneity of them. To resolve these problems, in this study we define the epigenetic modules as a gene set whose members are co-methylated and co-expressed. To address the heterogeneity of data, we construct gene co-expression and co-methylation networks, respectively. In this case, the epigenetic module is characterized as a common module in multiple networks. Then, a non-negative matrix factorization-based algorithm that jointly clusters the co-expression and co-methylation networks is proposed for discovering the epigenetic modules (called Ep-jNMF). Ep-jNMF is more accurate than the baselines on the artificial data. Moreover, Ep-jNMF identifies more biologically meaningful modules. And the modules can predict the subtypes of cancers. These results indicate that Ep-jNMF is efficient for the integration of expression and methylation data.

## 1. Introduction

DNA methylation modifies the cytosine base associating with cellular differentiation and cell development (Suzuki and Bird, 2008; Deaton and Bird, 2011; Teschendorff et al., 2012; Ziller et al., 2013). For example, DNA methylation regulates the expression of genes by decreasing the affinity of transcription factors (Bird and Wolffe, 1999). Furthermore, abberations of methylation directly result in oncogenesis of cancers (Varley et al., 2013). For instance, the methylation of CpG islands (CGIs) plays a critical role in renal cell cancers (Herman et al., 1994), breast cancer (Fleischer et al., 2014), and colorectal cancer (Hinoue et al., 2012).

Thus, it is promising to mine methylation patterns, such as the methylated CpG islands and epigenetic modules, because they are the foundation for revealing the mechanisms of cancers. For instance, dynamics of methylation of tissues is critical for the development of cells. The methylation patterns of genes closely associate with survival time of patients (Fleischer et al., 2014), and similarity of methylation profiles is also associated with cancer subtypes (West et al., 2013; Gavaert et al., 2015).

These efforts are insufficient to fully exploit the methylation patters because they only make use of methylation data, ignoring the regulation of methylation (Teschendorff and Relton, 2018; West et al., 2018). Since methylation directly regulates the expression of genes, it is natural to identify the epigenetic modules by integrating them. However, it is non-trivial for this issues largely due to two reasons. First, the pre-requisite of the integration of methylation and expression is the matched samples. Second, no cut-off definition of epigenetic modules is available because the regulation strategies vary. For instance, in most case, methylation in promoters negatively regulates the expression, whereas the positive regulation also exists (Varley et al., 2013).

For the first concern, the world consortia make use of the next-generation sequencing technologies to generate sample-matched data for cancers, which enables the possibility to exploit epigenetic modules. For instance, The Cancer Genome Atlas (TCGA)^1^ produces genomic data for various cancers, covering mutation, transcription, methylation, etc. Furthermore, Encyclopedia of DNA Elements (ENCODE)^2^ generate matched samples for cell lines and tissues.

For the second concern, even though it is intuitive to define epigenetic module for methylation profiles and networks by simply extending the traditional clustering problem, it is difficult to present a satisfied definition with heterogeneous data. The available algorithms for the integration of methylation and expression by either using a integrated network and multiple networks. Algorithms in the first class construct an integrated network, where the correlation between methylation and expression are integrate edge weight. Then, the epigenetic module in the integrated network is defined as a dense subgraph. For example, the FEM algorithm (Jiao et al., 2014) addresses this problem with the assumption that DNA methylation and expression is anti-correlated, where hot-spot and methylated modules are successfully identified. However, the recent evidence indicates that the correlation between methylation and expression could be both positive and negative (Varley et al., 2013), implying that the integrated network-based approaches are not precise enough to characterize the epigenetic modules.

To attack this issue, efforts have been devoted by using multiple networks to identify graph patterns. For example, in our previous study (Ma et al., 2014), dynamic modules are extracted from multiple networks by exploiting the temporality of cancer progression. Driver genes of cancers can be identified by exploiting the relations of various layers (Cantini et al., 2015), implying the importance and effectiveness of multiple networks. Clustering multiple networks aims to identify modules in networks, which can be achieved by extending measurement for single networks (Didier et al., 2015). These results demonstrate that multiple networks are more accurate and generalized than single networks in terms of characterizing biological patterns. In our previous study (Ma et al., 2017), the epigenetic module is a group of co-methylated and co-expressed genes in multiple networks, and then the epigenetic modules are discovered by using the M-Module algorithm (Ma et al., 2014). The success of the multiple network-based approaches demonstrates that the multiple networks model is much better than the integrated network base method.

**Figure 1:**
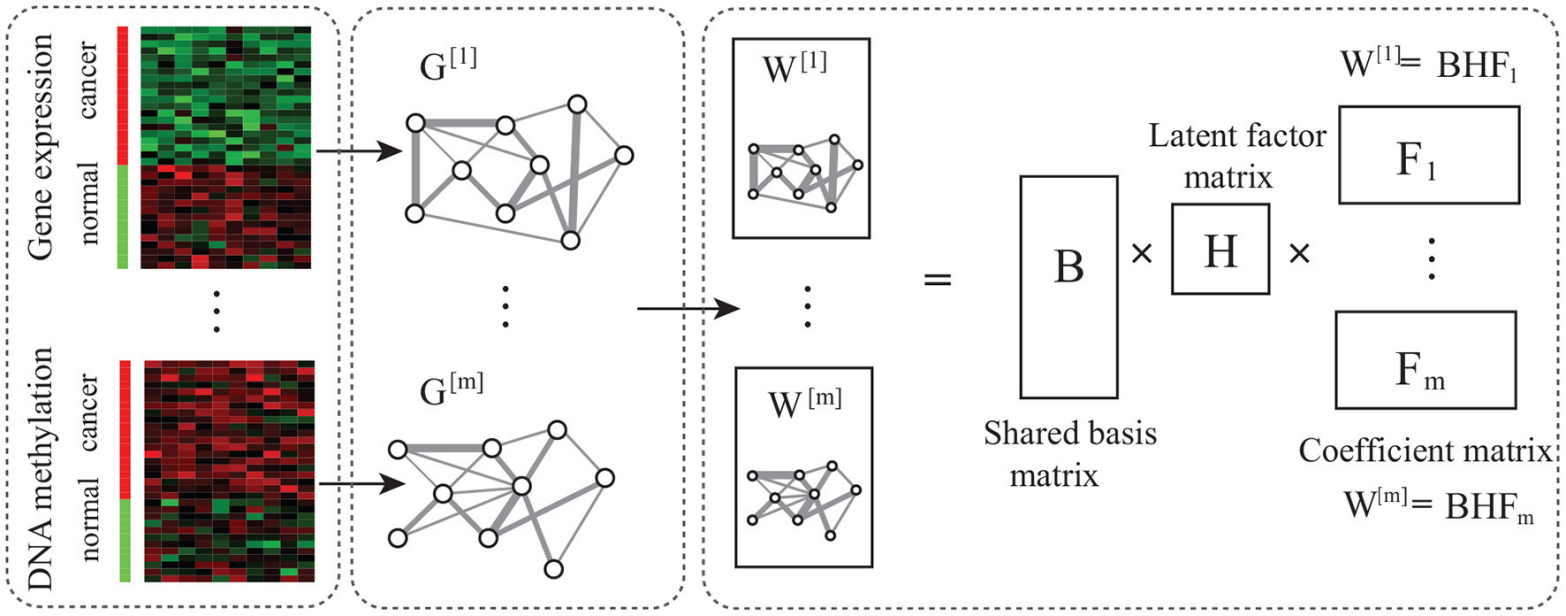
Overview of Ep-jNMF. It is composed of network construction, matrix factorization, and module discovery, where network construction obtains the gene co-expression and co-methylation networks, and the matrix decomposition extracts features.

Even though multiple network-based algorithms have been devoted to the epigenetic module discovery, many unsolved problems exit. Particularly, the quantification of modules in multiple networks is fundamental, and how to further improve performance of algorithms for epigenetic modules. In the present study, we discuss these two issues. To identify the epigenetic modules in the co-methylation and co-expression networks, the key problem is how to characterize the topological structure of modules in multiple networks. Then, we define the epigenetic module as the common module in multiple networks. To discover the functional epigenetic modules in multiple networks, a novel non-negative matrix factorization algorithm for epigenetic module (Ep-jNMF) is proposed, which jointly analyzes the gene co-expression and co-methylation networks (Figure 1). It first constructs the two layer networks, and extracts features using matrix factorization, where the topological structure is regularized into the objective function. Extensive experiments are performed, where Ep-jNMF achieves the best performance on the artificial networks. Moreover, it identifies more biological meaningful modules than the baselines, and some of obtained modules precisely predict the survival time of patients.

The rest of this study is organized as follows: section 2 presents the mathematical model and algorithm. The experiments and conclusion are depicted in sections 3 and 4, respectively.

## 2. Methods

The model and procedure of Ep-jNMF are depicted in this section.

### 2.1. Notations

A network (graph) is denoted by *G* = (*V, E*) with vertex set *V* and edge set *E*. Multiple network $G=\left\{G_{1},G_{2},\ldots ,G_{M}\right\}$ is a sequence of networks, where *G*_*m*_ is the *m*-th snapshot. In this study, the vertex set of G is fixed, i.e., *G*_*m*_ = (*V, E*_*m*_). The adjacent matrix of G is a tensor *W* = (_*w*_*ijm*_)*n*×*n*×*M*_, where *n* = |*V*| and *w*_*ijm*_ is the weight on the edge (*v*_*i*_, *v*_*j*_) in *G*_*m*_. Actually, *W* = [*W*_1_, *W*_2_, …, *W*_*M*_], where *W*_*m*_ = (_*w*_*ijm*_)*n*×*n*_ is the adjacency matrix of *G*_*m*_. In this study, the attached subscript *m* represents the value of the variable at condition *m*.

Vertex degree is the sum of weights on the incident edges, i.e., $d_{im}=\underset{j}{\sum }w_{ijm}$. Betweenness is a typical centrality (Freeman, 1979; Brandes, 2001), which is defined as

$$\begin{array}{l} betweenness_{m}\left(v\right)=\underset{v_{i}\neq v_{j},v_{i}\neq v,v_{j}\neq v}{\sum }\frac{g_{ivj}}{g_{ij}}, \end{array}$$

where *g*_*ivj*_ and *g*_*ij*_ are the number of the shortest paths between *v*_*i*_ and *v*_*j*_ passing, and without passing *v*, respectively. Given a group of genes, denoted by *C*, the density of *C* in network *G*_*m*_ is defined as

$$\begin{array}{l} Density_{m}\left(C\right)=\frac{2|E_{m}\left(C\right)|}{|C|\left(|C|-1\right)}, \end{array}$$

where *E*_*m*_(*C*) is the edge set of the subgraph induced by *C* in network *G*_*m*_, i.e., *E*_*m*_(*C*) = {(*v*_*i*_, *v*_*j*_)|*v*_*i*_∈*C, v*_*j*_∈*C*, (*v*_*i*_, *v*_*j*_)∈*E*_*m*_}.

**Figure 2:**
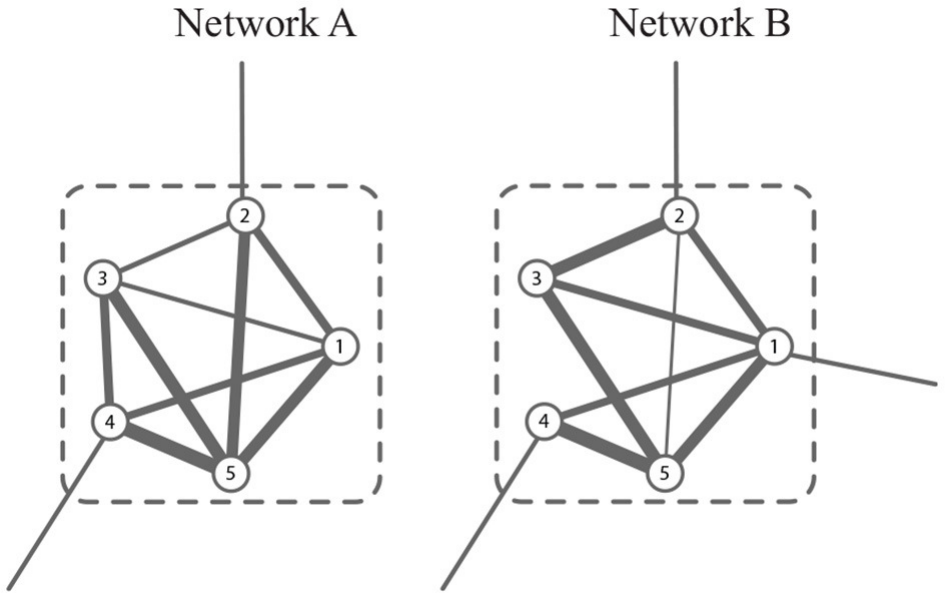
A schematic example of common module, which is well-connected in both networks.

In *G*, a module is a group of vertices with more edges within it, and fewer ones outside it. In G, the common module is a group of vertices whose connectivity is strong in all snapshots. For example, the module consisting of {1, 2, …, 6} in Figure 2 is well-connected in both networks. In this study, we aim to obtain the common modules in the co-expression and co-methylation networks. The common module detection corresponds to a hard partitioning {*C*_1_, *C*_2_, …, *C*_*k*_} (denoted by ${\left\{C_{l}\right\}}_{l=1}^{k}$) such that *C*_*l*_1__∩*C*_*l*_2__ = ∅ if *l*_1_≠*l*_2_ and $V=\underset{l}{\sum }C_{l}$, where *k* is the number of modules.

### 2.2. Mathematical Model

The quantification of connectivity of common modules in multiple networks is fundamental. Typical measurements, including the entropy function (Ma et al., 2014), modularity (Newman and Girvan, 2004), and modularity density (Li et al., 2008), are proposed. However, these strategies are inapplicable for the multiple networks. Here, we extend the modularity density *D* (Li et al., 2008) since it tolerates the resolution limit problem at some extent. Specifically, connectivity of module *C*_*l*_ in *G*_*m*_ is defined as

(1) $$\begin{array}{r} D_{m}\left(C_{l}\right)=\frac{1}{\sum_{v_{i}\in C_{l}}d_{im}}L\left(C_{l},C_{l}\right)-L\left(C_{l},{\bar{C}}_{l}\right), \end{array}$$

where $L\left(C_{l},C_{l}\right)=\underset{v_{i}\in C_{l},v_{j}\in C_{l}}{\sum }w_{ijm}$ and ${\bar{C}}_{l}=V\backslash C_{l}$. Ideally, we maximize the connectivity of module *C*_*l*_ in all snapshots, i.e.,

(2) $$\left\{ \begin{array}{l} \max D_{1}(\{C_{l}\}),\\ \;\cdots \\ \max D_{M}(\{C_{l}\}). \end{array}\right.$$

However, it is difficult to reach maximal value for each network. Therefore, we transform the multi-objective function in Equation (2) into a single objective function using the geometric mean of the connectivity, i.e.,

(3) $$\begin{array}{r} D\left(C_{l}\right)={\left(\underset{m}{\prod }D_{m}\left(C_{l}\right)\right)}^{1/M}. \end{array}$$

The underlying assumption is that a group of genes form a common module if and only if they are well-connected in all networks.

The partitioning ${\left\{C_{l}\right\}}_{l=1}^{k}$ is represented by *X*_*n*×*k*_ with *x*_*ij*_ =1 if *v*_*i*_∈*C*_*j*_, 0 otherwise. The overall function is the connectivity of all modules, i.e.,

(4) $$\begin{array}{r} \underset{l}{\sum }\max D\left(C_{l}\right) \end{array}$$

$$s.t.\left\{ \begin{array}{l} x_{ij}\in \{0,1\},\\ \sum_{j=1}^{k}x_{ij}=1,\\ \sum_{i=1}^{n}x_{ij}\geq 1, \end{array}\right.$$

where the second constraint enable the hard partitioning, and the last one ensures non-empty of modules. To avoid multi-objectives in Equation (4), we relax it as

(5) $$\begin{array}{r} \max\underset{l}{\sum }D\left(C_{l}\right) \end{array}$$

$$s.t.\left\{ \begin{array}{l} x_{ij}\in \{0,1\},\\ \sum_{j=1}^{k}x_{ij}=1,\\ \sum_{i=1}^{n}x_{ij}\geq 1, \end{array}\right.$$

### 2.3. The Ep-jNMF Algorithm

The algorithm consists of three components, which are introduced in turn (Figure 1). Networks are constructed using the Pearson correlation of gene profiles, and the PCIT package (Reverter and Chan, 2008) is adopted to remove noise.

NMF (Lee and Seung, 1999) approximates the target matrix using the product of two low-rank matrices as

(6) $$\begin{array}{r} W\approx BF \end{array}$$

$$s.t.\left\{ \begin{array}{l} B\geq 0,\\ F\geq 0, \end{array}\right.$$

where *B*_*n*×*k*_ and *F*_*k*×*n*_ are the basis and coefficient matrix, respectively, and *k* is the number of features. Usually, *k*≪*n* indicates that *BF* represents a compressed form of the original data *W*. Not allowing negative entries in *B* and *F* enables a non-subtractive combination of parts to form a whole. Equation (6) is solved by minimizing the approximation error as

(7) $$\begin{array}{r} e\left(B,F\right)=||W-BF|{|}^{2}, \end{array}$$

where ||*W*|| is the Frobenius Norm of matrix *W*. Tri-factorization is more efficient than NMF (Yoo and Choi, 2010), where Equation (8) is formulated as

(8) $$\begin{array}{r} e\left(B,F\right)=||W-BHF|{|}^{2}, \end{array}$$

where *H* is the factor matrix.

For each snapshot, Ep-jNMF jointly factorizes *W*_*m*_ as

(9) $$\begin{array}{r} W_{m}\approx BHF_{m}. \end{array}$$

Intuitively, we can minimize the approximation error for each snapshot as

(10) $$\begin{array}{r} \underset{m}{\sum }\min||W_{m}-BHF_{m}|{|}^{2} \end{array}$$

$$\text{s.t.}\left\{ \begin{array}{l} B\geq 0,\\ F_{m}\geq 0 \end{array}\right.$$

However, it is difficult to reach minimization for each snapshot. Similar to Equation (5), we reformulate Equation (11)

(11) $$\begin{array}{r} \min\underset{m}{\sum }||W_{m}-BF_{m}|{|}^{2} \end{array}$$

$$\text{s.t.}\left\{ \begin{array}{l} B\geq 0,\\ F_{m}\geq 0. \end{array}\right.$$

The algorithm iteratively updates *B* and *F*_*m*_ by following the multiplicative rules (Lee and Seung, 1999), where the update rules are formulated as

(12) $$\begin{array}{r} B=B\frac{\sum_{m}W_{m}F_{m}^{T}}{B\sum_{m}F_{m}F_{m}^{T}}, \end{array}$$

(13) $$\begin{array}{r} H=H\frac{\sum_{m}B^{T}F_{m}^{T}W_{m}}{B^{T}BF_{m}F_{m}^{T}}, \end{array}$$

and

(14) $$\begin{array}{r} F_{m}=F_{m}\frac{B^{T}W_{m}}{B^{T}W_{m}B}. \end{array}$$

Ep-jNMF (Algorithm 1) updates rules until termination is reached. For example, the approximation error threshold is set as 10^−2^, or the maximum iteration number is 10^3^. Because the initial solution is random, we repeat the procedure 50 runs with different initial solution matrices. The modules are extracted based on *B*, i.e., $x_{ij^{*}}=1$ where $j^{*}=\arg max_{j}B_{ij}$, 0 otherwise. The Ep-jNMF algorithm involves one parameter *k*, which is the number of features to obtain the coefficient matrices. We select it using the instability of matrix factorization (Wu et al., 2016).

**Table:** Algorithm 1 Ep-jNMF.

**Input:**
G: Networks;
*k*: Number of features;
**Output:**
${\left\{C_{l}\right\}}_{i=1}^{k}$: Common modules.
**Procedure I: network construction**
1: Constructing the gene co-expression (co-methylation) network using partial Pearson coefficient;
**Procedure II: matrix decomposition**
2: Fixing *F*_*m*_(1 ≤ *m* ≤ *M*) and *H*, update x *B* as equation (12);
3: Fixing *B* and *F*_*m*_(1 ≤ *m* ≤ *M*), update *H* as equation (13);
4: Fixing *B* and *H*, update *F*_*m*_(1 ≤ *m* ≤ *M*) as equation (14);
5: Keep updating the steps 3 and 4 until the termination criterion is reached;
**Procedure III: common module discovery**
6: Extracting modules from *B*;
7: **return**

### 2.4. Algorithm Analysis

On the space complexity, G requires space *O*(*n*^2^*M*). The basis matrix requires space *O*(*nk*) and the coefficient matrices need space *O*(*knm*). The space of the index matrix *X* is the same as the basis matrix *B*. In all, Ep-jNMF takes space *O*(*n*^2^*m*)+2*O*(*nk*)+*O*(*nkm*) = *O*(*n*^2^*M*) since *k*≪*n*.

On the time complexity, for each *F*_*m*_, Ep-jNMF needs time *O*(*rkn*^2^), where *r* is the number of iterations. And the running time for coefficient matrices in Ep-jNMF is *O*(*rkn*^2^*M*). Therefore, the total time complexity of Ep-jNMF is *O*(*rkn*^2^*M*).

## 3. Experiments

To validate the performance of Ep-jNMF, we select sixe state-of-the-art methods for a comparison, including M-Module (Ma et al., 2014), consensus clustering (CSC) (Cantini et al., 2015), multiple-modularity method (MolTi) (Didier et al., 2015), stability NMF (sNMF) (Wu et al., 2016), FEM (Jiao et al., 2014) and spectral clustering (SPEC) (Newman, 2006a), covering single-network- and multiple-network-based approaches. The former ones are extended using the consensus strategy (Cantini et al., 2015).

### 3.1. Data and Criteria

The artificial networks are derived from GN benchmark (Newman, 2006b), where each snapshot consists of 4 equal size communities with 32 vertices, and the degree of vertices is fixed as 16. Parameter *Z*_*out*_ controls the noise level of networks, and *Z*_*out*_ increases from 1 to 8. By manipulating parameter *Z*_*out*_, two types of multiple networks are generated, where in the homogeneous networks (HomoNet) the noise levels in snapshots are the same, and in heterogeneous networks (Heter-Net) it varies in different snapshots. Specifically, *Z*_*out*_ is fixed as 4 in the first snapshot, and it varies from 1 to 8 in the others. We downloaded the sample-matched gene expression and methylation profiles of breast cancer from TCGA. Specifically, the gene expression level is quantified using RPKM values and methylation level is measured by β signal, which are imputed using PCIT (Tibshirani et al., 2002).

The normalized mutual information (NMI) (Danon et al., 2005) measures the closeness of two partitioning: standard partition P^*^ and obtained partitioning *P*. NMI generates matrix *N* with the element *N*_*ij*_ as the size of vertices overlapped by $C_{i}^{*}$ and *C*_*j*_, which is formulated as

$$\begin{array}{l} NMI\left(P,P^{*}\right)=\frac{-2\sum_{i=1}^{\left|P\right|}\sum_{j=1}^{\left|P^{*}\right|}N_{ij}\log\left(\frac{N_{ij}N}{N_{i.}N_{.j}}\right)}{\sum_{i=1}^{\left|P\right|}N_{i.}\log\left(\frac{N_{i.}}{N}\right)+\sum_{i=1}^{\left|P^{*}\right|}N_{.j}\log\left(\frac{N_{.j}}{N}\right)}, \end{array}$$

where |*P*| is the cardinality of *P* and $N_{i.}=\sum_{j}N_{ij}$.

To check whether the predicted epigenetic modules are biological meaningful, various annotation databases are selected as gold standards for the enrichment analysis, where the significance is obtained by using the hypergeometric test (corrected by Benjamini–Hochberg test) with a cutoff of 0.05.

### 3.2. Performance on Simulated Networks

**Figure 3:**
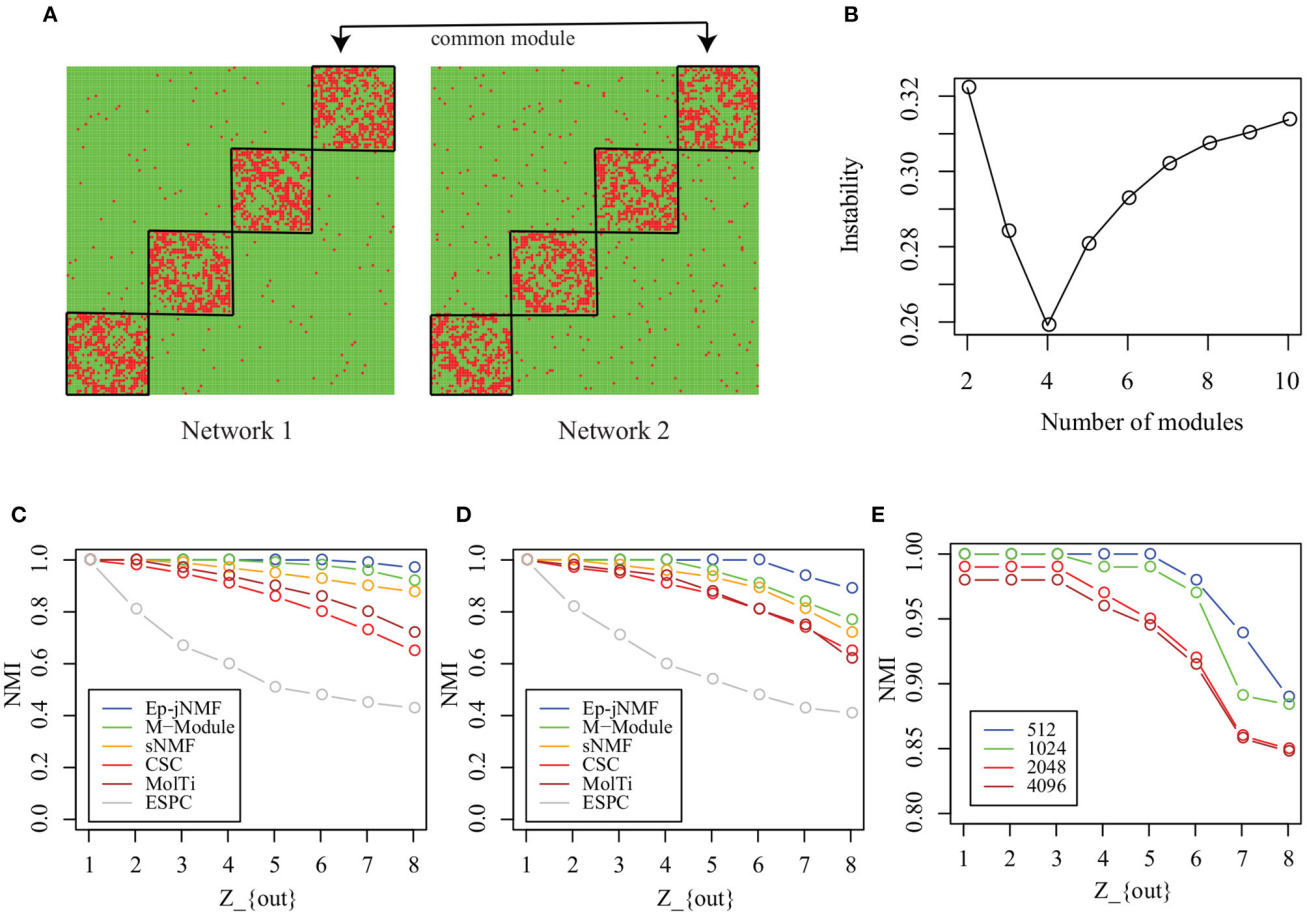
Performance on artificial networks: **(A)** Heatmap of common modules in Homo-Net (*Z*_*out*_ = 1); **(B)** selection of the number of modules using instability of matrix factorization with various noise levels; **(C)** NMIs of algorithms on Homo-Net; **(D)** NMI of algorithms on Heter-Net; and **(E)** scalability analysis of Ep-jNMF.

Each simulated snapshot contains 128 vertices and 4 modules of equal size with fixed degree 16. Parameter *Z*_*out*_ controls the noise level of networks. As *Z*_*out*_ increases from 1 to 8, the module structure is obscure. In this study, we generate two types of simulated networks with *M* = 2: Homo-Net and Heter-Net. Specifically, the parameter *Z*_*out*_ of both networks of Homo-Net is the same, while the *Z*_*out*_ of one network of Heter-Net is fixed as 4 and the parameter of the other network varies from 1 to 8. Figure 3A is the heatmap of the Homo-Net networks with *Z*_*out*_ = 1, where the common modules locate at the diagonal.

First, how the Ep-jNMF algorithm selects the parameter *k*, i.e., the number of modules, is studied. How the instability of Ep-jNMF changes as *k* increases from 2 to 10 for Homo-Net is shown in Figure 3B, where it chooses the optimal value 4 because the minimal is reached at 4. The similar pattern repeats for Heter-Net, which is not shown because of redundancy. The result demonstrates that the strategy is promising in selecting the number of modules.

Then, we compare M-Module, CSC, MolTi, sNMF, and SPEC on the simulated networks. Figure 3C shows the accuracy of various algorithms for Homo-Net, while Figure 3D shows the accuracy of various algorithms for Heter-Net. The performance of all these algorithms decreases as the parameter *Z*_*out*_ increases from 1 to 8 because the module structure is difficult to detect as *Z*_*out*_ increases. M-Module and Ep-jNMF outperform the rest of algorithms because the CSC, MolTi, and SPEC are based on the consensus clustering, which ignores the connection among multiple networks. However, M-Module and Ep-jNMF make use the multiple networks simultaneously during the module search procedure, which improves the accuracy of detecting the common modules. In all, the Ep-jNMF algorithm is better than the M-Module algorithm. More specifically, when *Z*_*out*_ is less than or equal to 5 in Homo-Net, the Ep-jNMF and M-Module algorithms have a similar performance. When *Z*_*out*_ is greater than or equal to 6, Ep-jNMF outperforms M-Module, indicating the superiority of Ep-jNMF. The similar tendency also repeats in Heter-Net (Figure 3D).

Finally, we investigate the accuracy of Ep-jNMF by increasing the number of vertices from 512 to 4096. The performance of Ep-jNMF is shown in Figure 3E, suggesting that the algorithm is robust. These results demonstrate that Ep-jNMF is promising to identify common modules in artificial networks.

### 3.3. Performance on Cancer Networks

For cancer networks, we select the Ep-jNMF, M-Module, MolTi, sNMF, and FEM algorithms for a comparison since they significantly outperform CSC and SPEC. The Ep-jNMF, M-Module, MolTi, sNMF, and FEM algorithms identify 17, 26, 94, 26, and 460 modules, respectively.

**Figure 4:**
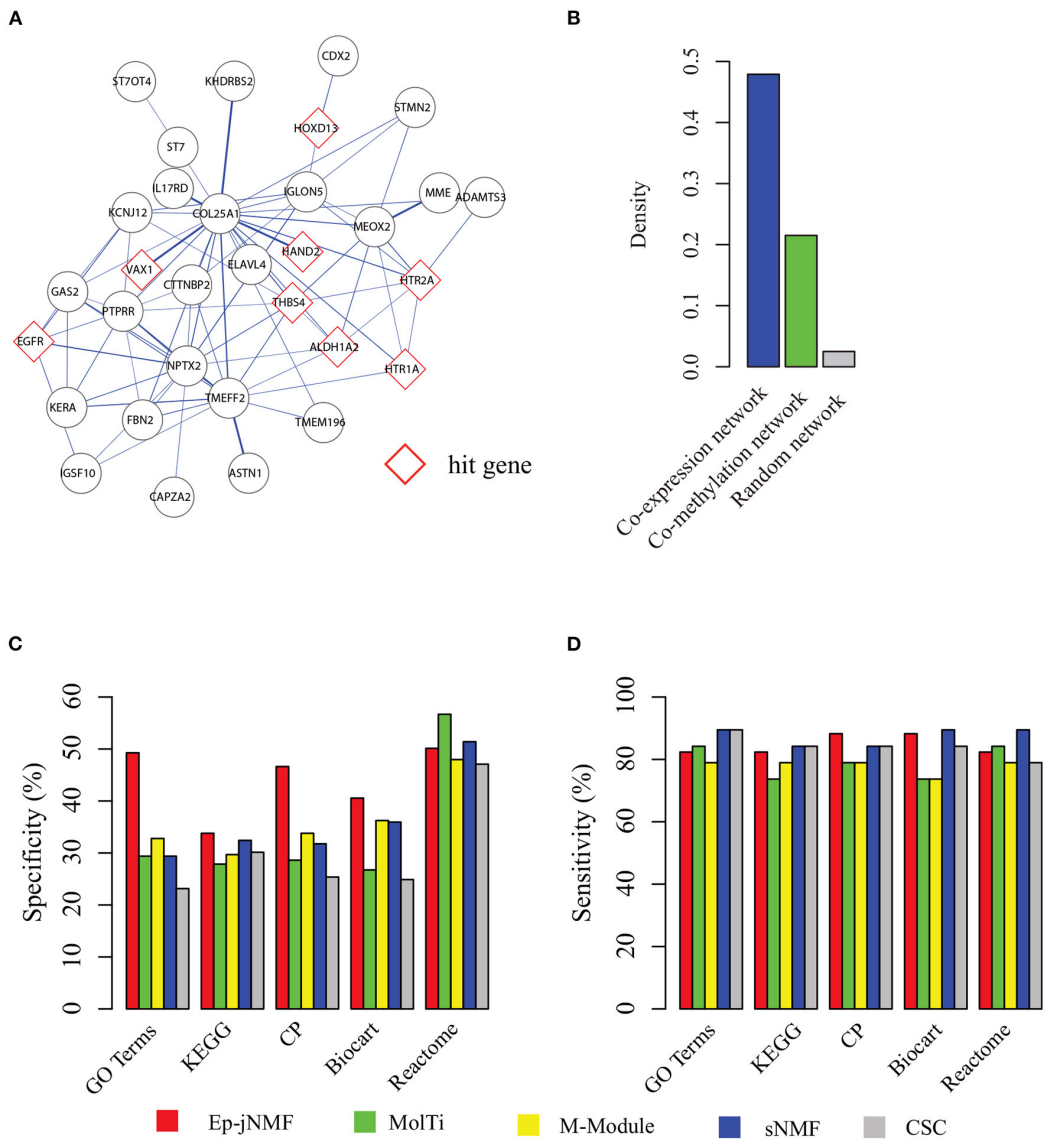
Performance on the cancer networks: **(A)** a typical epigenetic module predicted by Ep-jNMF with red border color as hit genes; **(B)** the density of the modules in the co-methylation and co-expression network; **(C)** percentage of predicted modules enriched by one reference pathways or functions (specificity); and **(D)** percentage of reference pathways or functions enriched by a predicted module.

Figure 4A presents a functional epigenetic module obtained by Ep-jNMF with cell proliferation (p = 3.8E-4), which is critical for breast cancer metastasis (Loayza-Puch et al., 2016; Thienpont et al., 2016). Interestingly, the epigenetic module contains the HAND2 sub-module, which is validated by the biological experiments (Jones et al., 2013). The HAND2 module has been used as the benchmark for the algorithms for the methylated module (Jiao et al., 2014). Furthermore, we find that only FEM and Ep-jNMF can discover the HAND2 module, whereas the others cannot. These results imply that Ep-jNMF is effective for the identification of critical epigenetic modules. To check whether the genes within the obtained common module are well-connected in both networks, the density of the module in different snapshots is shown in Figure 4B. Clearly, the connectivity is strong in both snapshots because the density is 0.47 and 0.22, which is significantly higher than that in random networks. The possible reason why the module is much denser in the co-expressed network than that in the co-methylated network is that methylation is more specific than expression.

To fully validate the performance of Ep-jNMF, Gene Ontology (Ashburner et al., 2000), KEGG (Kanehisa et al., 2012), Reactome (Croft et al., 2014), Biocart (Nishimura, 2001), and Canonical pathways (Subramanian et al., 2005) are selected as reference annotation. To evaluate the performance, we first check the percentage of predicted modules that significantly enriched by at least one reference annotation, and then we calculate the percentage of the reference pathways that significantly overlaps with at least one predicted module. Figures 4C,D show that Ep-jNMF achieves higher specificity with comparable sensitivity, implying that the predicted modules are more meaningful in terms of the biological background.

### 3.4. Performance on Predicting Cancer Subtypes

**Figure 5:**
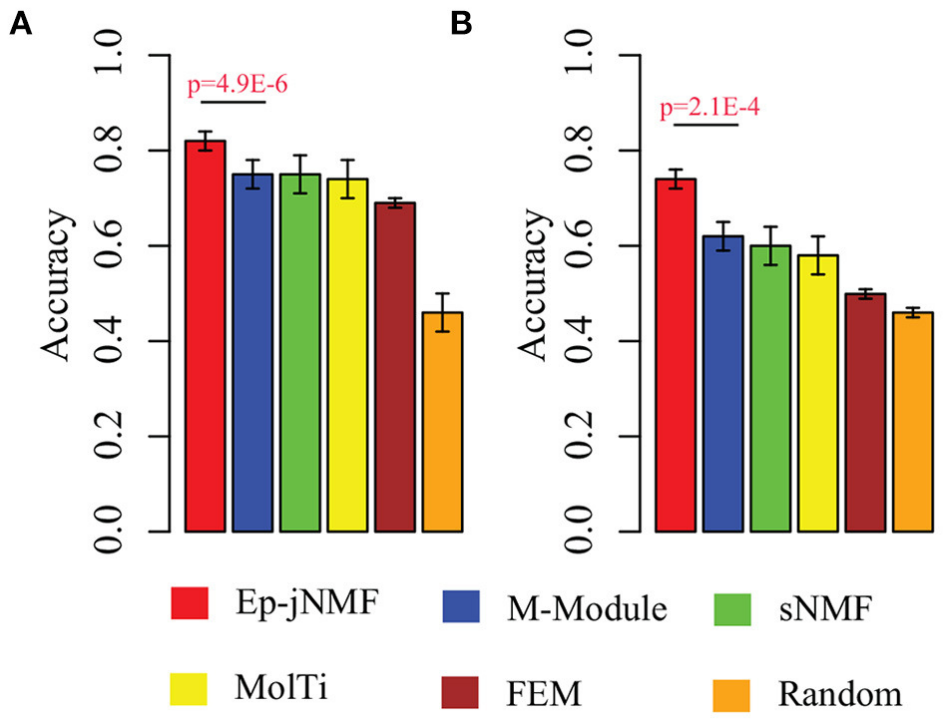
Performance on the prediction of subtypes with various validation in terms of accuracy: **(A)** internal validation with the error bar as for the standard deviation and **(B)** external validation.

Evidence proves that hub genes facilitate the prognosis of cancers (Taylor et al., 2009). Therefore, we check whether epigenetic modules also serve as biomarkers to discriminate cancer subtypes by using the methylation profiles. We select modules predicted by Ep-jNMF, FEM, sNMF, M-Module, and MolTi. Furthermore, we also include size-matched set of randomly modules to validate the performance of different features. Support vector machine is selected as classifier to calculate the percentage of patient samples that are classified correctly (accuracy). The fivefold cross-validation is used for SVM, which is shown in Figure 5A, indicating that modules obtained by Ep-jNMF are more discriminative than the others. Specifically, the accuracy of Ep-jNMF is 82.4%, whereas that of M-Module is 75.1% (*p* = 4.9E-6, Wilcoxon test), showing that modules in multiple networks are more accurate to capture the structure and functions of cancers. The external dataset is also performed (GSE5874), which is shown Figure 5B. Specifically, Ep-jNMF is also superior to the baselines (i.e., 74.6% for Ep-jNMF vs. 62.9% for M-Module, *p* = 2.1E-4, Wilcoxon test).

## 4. Conclusion

Epigenetic modification is a critical biological process, and mining the patterns is promising for the understanding of cancers. The advances in the next-generation sequencing technologies facilitate the generation of genomic data for cancers, which enables the integrative analysis of omic data. How to integrate gene methylation and expression data is the fundamental step for revealing the mechanisms of cancers. The traditional methods fuse them into a single network by assuming the positive and negative correlation between expression and methylation. However, these strategies are criticized for the undesirable performance since the underlying assumption is not consistent with the biological principle.

In this study, we use the multiple networks model to characterize functional epigenetic modules, which corresponds to the common modules detection in multiple networks. Finally, we present a matrix factorization algorithm for extracting the common modules from heterogeneous networks. Overall, the contributions are summarized as follows: (i) it provides a mathematical model for the functional epigenetic modules, which overcomes the limitation of the current approaches, i.e., the correlation specification between methylation and expression is not required; (ii) a joint learning method is proposed to identify the epigenetic modules in multiple networks, which avoids the structure preservation of single network-based method, which can be easily extended for other data, such as Chip-seq and mutation data; and (iii) the experiments show the superiority of Ep-jNMF.

In further research, we will investigate how to integrate heterogeneous entities, such as microRNAs, to extract the regulation programming based on multiple heterogeneous networks.

## Data Availability Statement

Publicly available datasets were analyzed in this study. This data can be found here: TCGA.

## Author Contributions

ZD and XM designed the method and coded the algorithm. XM wrote the paper. Both authors contributed to the article and approved the submitted version.

## Conflict of Interest

ZD is employed by China Electronics Technology Group Corporation. The remaining author declares that the research was conducted in the absence of any commercial or financial relationships that could be construed as a potential conflict of interest.

## Publisher's Note

All claims expressed in this article are solely those of the authors and do not necessarily represent those of their affiliated organizations, or those of the publisher, the editors and the reviewers. Any product that may be evaluated in this article, or claim that may be made by its manufacturer, is not guaranteed or endorsed by the publisher.

## Footnotes

^1^ https://cancergenome.nih.gov/

^2^ https://www.encodeproject.org/
